# Field assessment of genome‐edited, low asparagine wheat: Europe's first CRISPR wheat field trial

**DOI:** 10.1111/pbi.14026

**Published:** 2023-02-21

**Authors:** Sarah Raffan, Joseph Oddy, Andrew Mead, Gary Barker, Tanya Curtis, Sarah Usher, Christopher Burt, Nigel G. Halford

**Affiliations:** ^1^ Rothamsted Research Hertfordshire UK; ^2^ Functional Genomics, School of Biological Sciences University of Bristol Bristol UK; ^3^ Curtis Analytics Limited Kent UK; ^4^ RAGT Seeds Ltd. Essex UK

**Keywords:** Wheat, CRISPR‐Cas9, field trial, acrylamide, asparagine synthetase

We reported in this journal in 2021 the generation of wheat genotypes in which the asparagine synthetase gene, *TaASN2*, had been ‘knocked out’ using CRISPR‐Cas9 (Raffan *et al*., 2021). The editing had been achieved by introducing genes encoding the Cas9 nuclease, four guide RNAs (gRNAs) and a *Bar* marker gene into wheat (*Triticum aestivum*) cv. Cadenza. Here we report the results of a field trial of Line 178.35, an A genome null for *TaASN2*, and total nulls, 23.60 and 23.75 (Raffan *et al*., 2021). Also included were four AB genome nulls, referred to as TILLING lines 1–4, derived from a selected line of a mutant population produced by ethyl methanesulphonate treatment of wheat cv. Cadenza seeds (Rakszegi *et al*., 2010). The mutated *TaASN2‐A2* gene from this line was backcrossed into the cv. Claire background to generate AB genome nulls (cv. Claire lacks a B genome *TaASN2* gene due to a ‘natural’ deletion (Oddy *et al*., 2021)).

The gRNA and *Bar* genes were segregating in all three GE lines, whilst only line 23.60 still contained the *Cas9* gene. Consent to release the GMOs was granted by the UK government on 31/08/2021 (reference 21/R08/01). To our knowledge, the field trial was the first in Europe to include genome‐edited (GE) wheat lines. A second total null GE line that had been produced, Line 59 (Raffan *et al*., 2021), was found to have additional edits in the closely related gene, *TaASN1*. Because these additional edits were not described in the application for consent to release the GMOs, this line was not included in the field trial.

The aim of knocking out *TaASN2* was to reduce the concentration of free asparagine in the grain. Free asparagine is converted to the carcinogenic processing contaminant, acrylamide, during high‐temperature processing, baking and toasting (Raffan and Halford, 2019). The concentrations of free asparagine in the grain of line 178 plants grown under glass were 56% and 68% of controls over two generations, while those of line 23 were 43% and 57% of controls. The aim of the field trial was to determine whether the low asparagine phenotype was maintained under field conditions and assess the performance of the lines with respect to emergence, yield, thousand grain weight (TGW) and composition.

The field trial was sown at the Rothamsted farm, Harpenden, UK, on 26/10/2021. It comprised 56 plots of 6 × 1.8 m (Figure 1a), with five plots of each of GE lines 178.35, 23.60 and 23.75, TILLING lines 1–4, a TILLING control, which had come through the backcross process but contained no mutations in *TaASN2*, plus 8 plots each of Cadenza and Claire. A test prior to drilling showed a germination rate of >95% for all of the lines, and all plots showed emergence by 01/12/2021.

**Figure 1:**
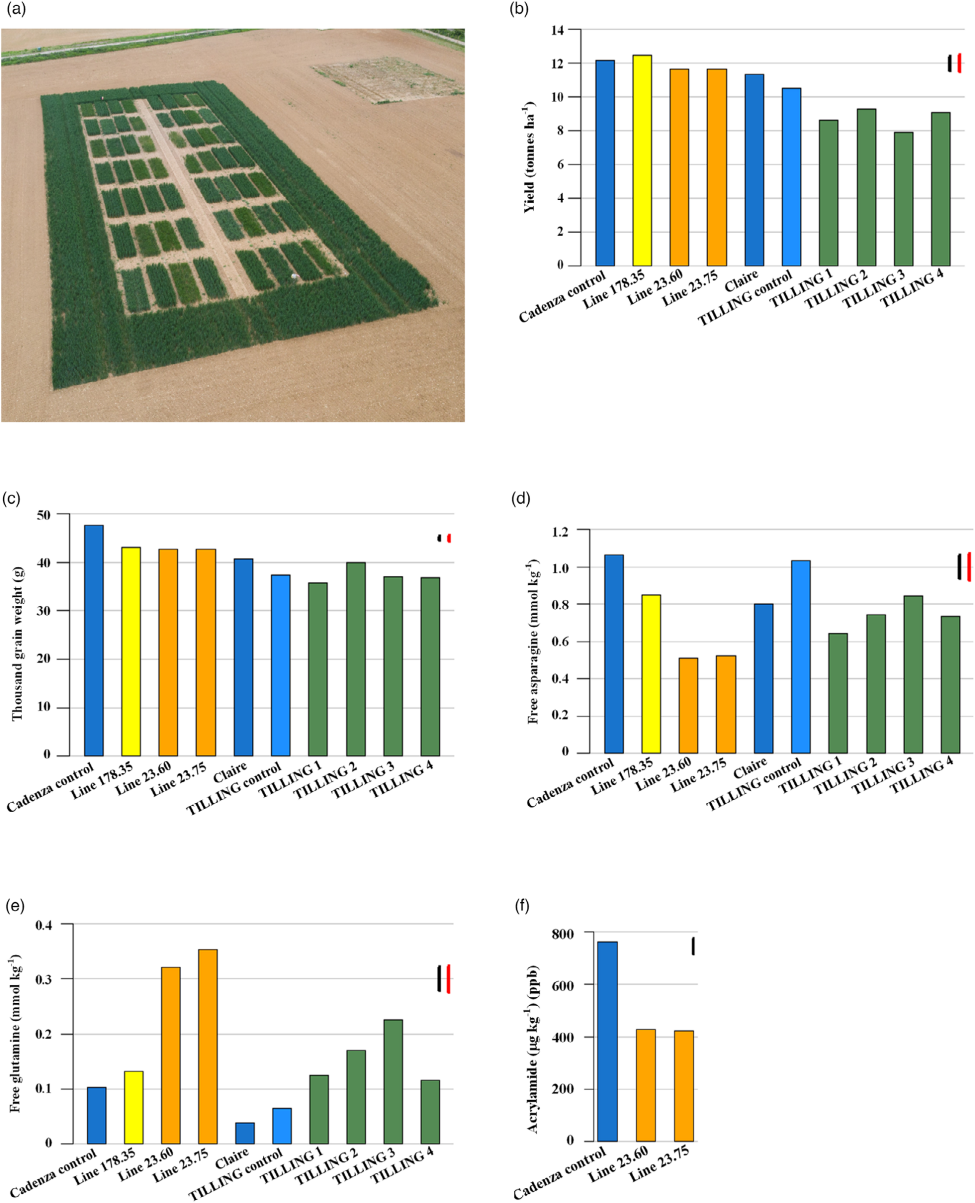
(a) Aerial shot of the field trial, showing the layout of the 56 plots and the pollen barrier. (b, c) Mean yield and TGW for the different lines. (d, e) Mean free asparagine and glutamine concentrations in flour prepared from the different lines. (f) Mean acrylamide formed in heated flour from plots of control Cadenza and GE Lines 23.60 and 23.75. For b–e, LSD (5%) is indicated by two lines, one (black) for comparisons between the mutant genotypes (TILLING and GE) and the controls (Claire and Cadenza), the other (red) for comparisons within the mutant genotypes (TILLING and GE). For f, a single LSD (5%) is shown.

## Yield

The trial was harvested on 12/08/2022. There were significant differences in yield (*F*_9,33.7_ = 25.46, *p* < 0.001) (Figure 1b; Data S1), with the TILLING lines performing worse than Claire (*F*_1,29.9_ = 58.82, *P* < 0.001). This may reflect the effect of background mutations still present in these lines. In contrast, there were no significant differences between the GE lines and Cadenza (*F*_1,31.5_ = 0.95, *P* = 0.337), illustrating the obvious advantage of the targeted GE approach compared with random mutagenesis. There were also significant differences in TGW between the lines (*F*_9,36.6_ = 113.63, *P* < 0.001) (Figure 1c), with the GE lines showing a 10% reduction overall compared with Cadenza (*F*_1,35.8_ = 167.41, *P* < 0.001), meaning that they produced more but smaller grains. We speculate that perturbations of asparagine metabolism affected seed set, with the increased number of seeds in the GE lines being compensated for by reduced resource allocation to each seed, but this requires further investigation. Importantly, the GE seed size was well within the normal range for wheat. The TILLING lines showed reductions from 2.5 to 12% compared with Claire (*F*_1,34.9_ = 95.22, *P* < 0.001), although there was no difference between the control and knockout TILLING lines (*F*_1,37.3_ = 0.04, *P* = 0.847).

## Free amino acid concentrations

Free amino acid concentrations in flour prepared from the harvested grain were measured as described previously (Raffan *et al*., 2021). The predicted means for total and individual free amino acid concentrations and statistical analyses are given in Data S1.

There were significant differences in the free asparagine concentration of the different lines (Figure 1d) (*F*_9,35.3_ = 14.92, *P* < 0.001). The GE lines showed a significant reduction (*F*_1,34.8_ = 74.95, *P* < 0.001), with the mean concentrations in the two L23 lines at just under 50% that of Cadenza and Line 178 at 86%. The GE lines also showed significant increases in free glutamine (*F*_1,30.7_ = 102.49, *P* < 0.001) (Figure 1e) (the amino group transferred to aspartate to make asparagine comes from glutamine).

The TILLING lines showed more variable responses when compared to Claire, with one (TILLING 3) showing an increase in free asparagine and the others a small decrease (Figure 1d). However, when compared with the TILLING control, they did show significant reductions of 20–40% (*F*_1,35.7_ = 26.57, *P* < 0.001), comparable with the reduction of 28% seen previously in A genome TILLING nulls in the Cadenza background, and 24–34% in the tetraploid Kronos background (Alarcón‐Reverte *et al*., 2022). There was also an increase in free glutamine in the TILLING lines when compared with Claire (*F*_1,29.3_ = 47.97, *P* < 0.001) or the TILLING control (*F*_1,37.0_ = 23.71, *P* < 0.001) (Figure 1e).

## Other aspects of grain composition

There was no difference between the lines in nitrogen or carbon content (Data S1). However, there were differences in the sulphur content (*F*_1,46.0_ = 594.90, *P* < 0.001), with the GE lines showing significantly higher grain sulphur than Cadenza (*F*_1,46.0_ = 594.90, *P* < 0.001). The TILLING mutant lines also showed a higher concentration than their control (*F*_1,46.0_ = 172.65, *P* < 0.001).

## Acrylamide in heated flour

Flour (1.0 g) produced from grain from the five plots of each of Lines 23.60 and 23.75 and five Cadenza plots was heated to 160°C, and acrylamide content was analysed by liquid chromatography–tandem mass spectrometry. The method used was compatible with the CEN standard.

Acrylamide formed in the Cadenza flour to an average concentration of 761 μg/kg (parts per billion) (Figure 1f), while the average concentrations in Lines 23.60 and 23.75 were 427 and 421 μg/kg, respectively, representing decreases of 44–45% compared with Cadenza (*P* < 0.001).

## Conclusions

The study showed that step reductions in the free asparagine concentration of wheat grain achieved using genome editing are maintained in the field, with a concomitant effect on acrylamide formation in heated flour and with no significant effects on yield or nitrogen content, at least within this single year/site trial. This is important because the availability of low acrylamide wheat could enable food businesses to comply with evolving regulations on acrylamide without costly changes to production lines or reductions in product quality. It could also have a significant impact on dietary acrylamide intake for consumers. However, GE plants will only be developed for commercial use if the right regulatory framework is in place and breeders are confident that they will get a return on their investment in GE varieties.

## Conflict of interest

The authors had complete control of the design of the study, the collection, analysis and interpretation of data, the writing of the manuscript and the decision to publish, without interference from the funders and partner organizations listed in the acknowledgements.

## Author contributions

SR conducted the experiments and analysed the data with JO; AM was responsible for statistical analysis of the data; TYC and SU produced the data on free amino acid concentrations in the grain and acrylamide formation in flour; CB produced the TILLING lines used in the study; GB and NGH were the project leaders and together with SR were responsible for the conception and design of the study; SR and NGH drafted the manuscript.

## Supporting information


**Table S1.** Predicted means and standard errors of the differences for free amino acid and mineral concentrations, %N and %C, for wheat varieties Cadenza and Claire, genome edited lines 178.35, 23.60 and 23.75, TILLING lines 1‐4, and a TILLING control.
**Table S2.** REML variance components analysis of the data for free amino acid and mineral concentrations, %N and %C.
**Table S3.** Analysis of variance of the acrylamide data for heated flour from Cadenza and genome edited lines 23.60 and 23.75.
